# Resection quality and oncologic outcomes after robotic versus laparoscopic total mesorectal excision for mid and low rectal cancer: a systematic review and meta-analysis of randomised trials

**DOI:** 10.1007/s11701-026-03541-z

**Published:** 2026-06-01

**Authors:** Rathin Gosavi, Baxter Smith, Anna Mealy, Wafa Iftekher, Stephen Bell, Paul McMurrick, Thang Chien Nguyen, William Teoh, Vignesh Narasimhan

**Affiliations:** 1 Department of Colorectal Surgery, Cabrini Health, Melbourne, VIC Australia; 2 Department of Colorectal Surgery, Monash Health, Melbourne, VIC Australia; 3 Department of Surgery, School of Clinical Sciences at Monash Health), Monash University, Melbourne, VIC Australia; 4 Department of Colorectal Surgery, Alfred Health, Melbourne, VIC Australia

**Keywords:** Robotic surgery, Robotic total mesorectal excision, Laparoscopic total mesorectal excision, Total mesorectal excision completeness

## Abstract

Total mesorectal excision (TME) for mid and low rectal cancer is technically demanding, and resection quality strongly influences local control and survival. Robotic platforms may offer technical advantages over conventional laparoscopy, but oncologic benefits remain uncertain. This meta-analysis evaluates resection quality and early oncologic outcomes using evidence exclusively from randomised trials. A PRISMA aligned systematic review of MEDLINE, Embase, CENTRAL and Google Scholar was performed to 22 September 2025. Eligible studies were parallel-group randomised controlled trials comparing robotic with laparoscopic TME for mid or low rectal adenocarcinoma, defined as tumour height of 10 cm or less from the anal verge. Co-primary outcomes were circumferential resection margin (CRM) positivity and completeness of the mesorectum. Secondary outcomes included conversion to open surgery, intraoperative complications, and three-year locoregional recurrence, disease-free survival, and overall survival. Odds ratios and hazard ratios were pooled using random effects models. Four randomised trials met inclusion, enrolling 1,952 patients. Robotic TME reduced CRM positivity (OR 0.58, 95% CI 0.38 to 0.87) and increased complete TME rates (OR 1.55, 95% CI 1.14 to 2.12). Conversion to open surgery was less frequent with robotics (OR 0.41, 95% CI 0.21 to 0.79). Intraoperative and early postoperative complications did not differ. Two trials reported three-year oncologic outcomes. Robotic TME was associated with lower three-year locoregional recurrence (OR 0.43, 95% CI 0.23–0.81) and a modest improvement in disease-free survival (HR 0.78, 95% CI 0.61–0.99), although event rates were low and estimates should be interpreted cautiously. Overall survival did not differ (HR 0.79, 95% CI 0.57–1.11). In mid and low rectal cancer, robotic TME was associated with improved pathological resection quality and lower conversion rates compared with laparoscopic TME, without clear differences in early perioperative morbidity There is emerging evidence of improved three-year locoregional recurrence and disease-free survival.

**Supplementary Information:**

The online version contains supplementary material available at 10.1007/s11701-026-03541-z.

## Introduction

Total mesorectal excision (TME) remains the cornerstone of curative surgery for rectal cancer, with the quality of the mesorectal specimen and the status of the circumferential resection margin (CRM) recognised as critical determinants of local control and long-term survival [1–3]. Achieving these oncologic benchmarks is considerably more challenging in mid and low rectal tumours, where pelvic confinement, bulky disease, prior chemoradiotherapy, android pelvis, obesity and a restricted working field amplify the technical complexity of dissection [4–6]. In this setting, conversion to open surgery is not a benign event: it typically reflects intraoperative difficulty, may compromise recovery, and represents a significant deviation from the intended benefits of minimally invasive surgery [7, 8].

Robotic platforms were developed in part to overcome the well-documented limitations of conventional laparoscopy in the pelvis. Key features such as stable three-dimensional vision, wristed instrumentation, and tremor filtration offer potential advantages in confined spaces, particularly in maintaining dissection planes and facilitating atraumatic mesorectal mobilisation [9, 10]. Whether these technical attributes confer meaningful improvements in oncologic surgery has, however, remained uncertain. Much of the existing literature has been confounded by heterogeneity in tumour height, procedural type, and study design, with prior meta-analyses frequently dominated by non-randomised cohorts and surrogate endpoints [11, 12]. Early randomised trials often emphasised perioperative metrics over audited pathology, and few were adequately powered or sufficiently mature to report time-to-event oncologic outcomes [9, 13, 14].

Randomised controlled trials (RCTs) directly comparing robotic and laparoscopic TME have now matured and increasingly report standardised oncologic endpoints. Standardised TME quality assessment and CRM involvement are routinely assessed, and some trials now include long-term outcomes such as disease-free and overall survival at three years [10, 15]. Importantly, several recent RCTs have limited inclusion to mid and low rectal tumours, where any benefit of robotic instrumentation is likely to be most apparent [13–16]. This more refined evidence base enables a targeted synthesis that better reflects both anatomical challenge and contemporary surgical standards.

This systematic review and meta-analysis evaluates the comparative effectiveness of robotic versus laparoscopic TME specifically for mid and low rectal adenocarcinoma, using data exclusively from randomised trials. The primary outcomes are resection quality defined by TME quality and CRM status. Secondary outcomes include conversion, three-year oncologic endpoints and major morbidity, to assess both technical performance and longer-term surgical adequacy. By focusing on this anatomically distinct cohort and high-quality evidence, this review aims to clarify the oncologic role of robotic TME in routine rectal cancer surgery.

## Methods

### Eligibility criteria

Eligible studies were parallel group RCTs that enrolled adults with mid or low rectal adenocarcinoma undergoing curative resection. Mid or low rectum was defined as a tumour height of 10 cm or less from the anal verge. The intervention was robotic TME and the comparator was laparoscopic TME. Trials of TME without a robotic versus laparoscopic comparison, non-randomised studies, mixed colorectal cohorts without separable rectal data, and studies confined to upper rectal cancers were excluded. Trials were required to report at least one of intraoperative complications, histopathological and conversion outcomes.

### Outcomes

The co-primary outcomes were circumferential resection margin (CRM) positivity, defined as tumour at or within 1 mm of the inked radial margin, and mesorectal quality. Mesorectal quality was reported in all included trials using a three-tier grading framework (complete, nearly complete, incomplete), consistent with the established plane-of-surgery (Quirke-type) assessment of the TME specimen [17, 18]; to permit synthesis, we analysed mesorectal quality as complete versus non-complete, with non-complete comprising nearly complete and incomplete grades.

Secondary outcomes were deliberately restricted to three domains: conversion to open surgery, perioperative complications, and long-term oncologic outcomes. Conversion was defined as unplanned open surgery (laparotomy) as reported by each trial; where specified, this included an incision larger than that required for specimen extraction. Intraoperative complications were analysed as a trial-defined composite of adverse events occurring during the index operation. Postoperative complications were analysed as trial-defined overall postoperative morbidity Long-term oncologic outcomes comprised three-year locoregional recurrence, disease-free survival, and overall survival. Locoregional recurrence was extracted at approximately three years as reported by each trial. Disease-free and overall survival were extracted as hazard ratios where reported or derived as described below.

### Search strategy and study selection

**Fig. 1:**
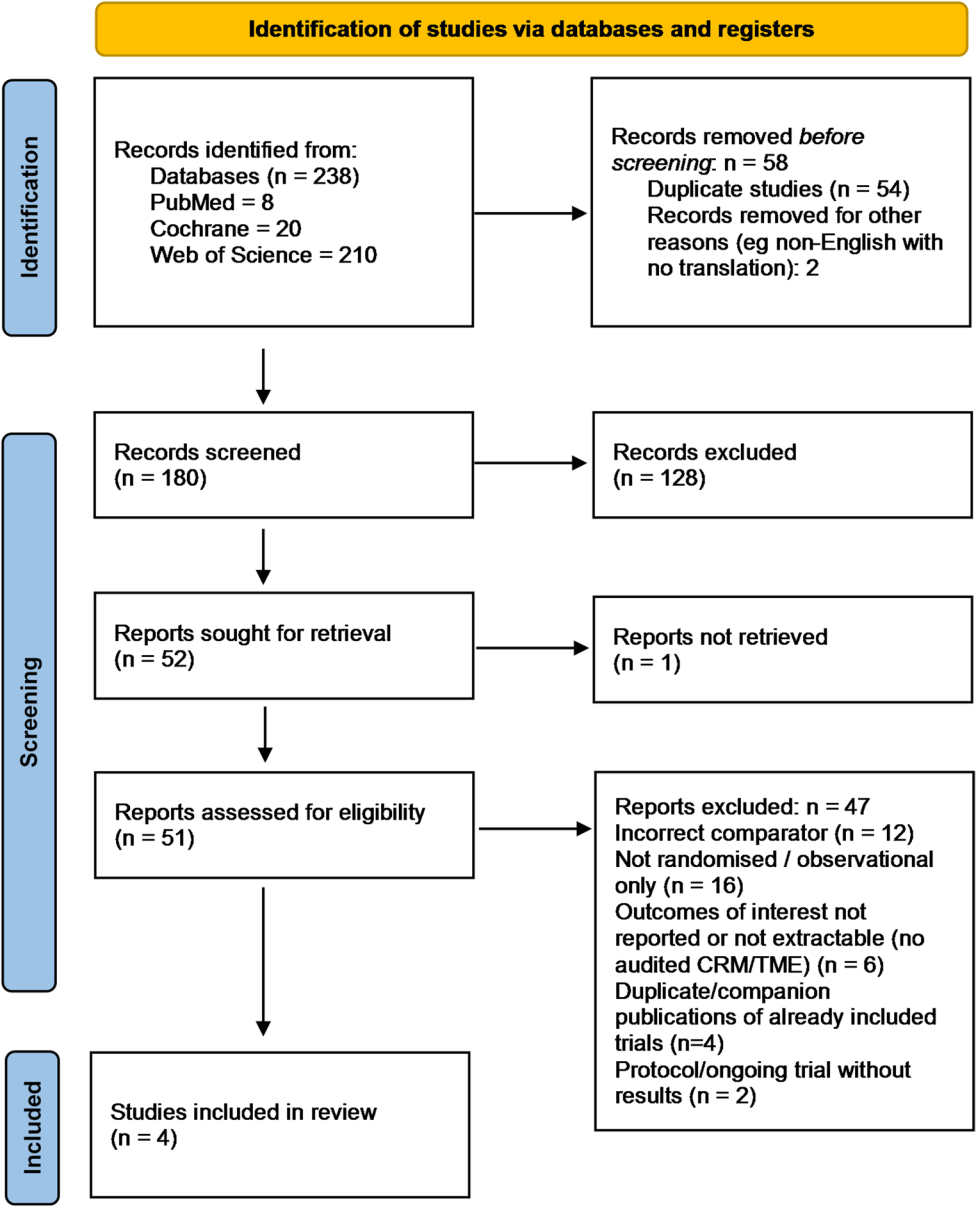
PRISMA search

A comprehensive search of MEDLINE, Embase, Google Scholar and the Cochrane Central Register of Controlled Trials (CENTRAL) was performed from inception to 22/09/2025 and reported in accordance with PRISMA 2020. Additional records were identified through manual searching of reference lists, trial registries (ClinicalTrials.gov), and relevant systematic reviews. No language restrictions were applied. Two reviewers (RG and BS) independently screened all titles, abstracts, and full texts for inclusion, with discrepancies resolved through discussion with the senior author (VN). Full database-specific search strategies are provided in the Supplementary material (Supplementary Table 1), and the PRISMA flow diagram is presented in Fig. 1. A completed PRISMA 2020 checklist is provided in the Supplementary material.

### Data extraction

Two reviewers (RG and BS) independently extracted trial design, setting, eligibility definitions, baseline characteristics, operative details, and outcome data. For dichotomous outcomes we captured events and totals by arm. For continuous outcomes we recorded means and standard deviations. Where only medians and interquartile ranges were available, we estimated means and standard deviations using validated distribution-based methods [19]. For time-to-event outcomes we extracted reported hazard ratios with confidence intervals. If hazard ratios were not reported, we derived log hazard ratios and standard errors from Kaplan–Meier curves using established reconstruction methods [20, 21]. Trial authors were contacted for clarification where necessary.

### Risk of bias and certainty assessment

Risk of bias was assessed at the outcome level using the Cochrane RoB 2.0 tool across domains of randomisation, deviations from intended interventions, missing outcome data, outcome measurement, and selection of reported results. Overall judgements followed Cochrane guidance, whereby ‘some concerns’ in any domain generally resulted in an overall judgement of ‘some concerns’ for that outcome. Certainty of evidence for each outcome was rated with GRADE, considering risk of bias, inconsistency, indirectness, imprecision, and potential publication bias.

### Effect measures and synthesis

Dichotomous outcomes (CRM positivity, complete TME, conversion, intraoperative complications, and locoregional recurrence when reported as proportions) were pooled as odds ratios with random effects. Time-to-event outcomes (DFS and OS, and LRR when reported as hazard ratios) were synthesised as log hazard ratios with standard errors using generic inverse variance. When trials reported LRR only as cumulative incidence at three years, we used odds ratios and performed a sensitivity analysis against any reported hazard ratio. Where definitions varied (particularly for complications), outcomes were extracted and pooled using trial-reported definitions.

For the Summary of Findings table, absolute risks were derived from the pooled baseline risk in the laparoscopic (control) arms of the trials contributing to each outcome. Absolute effects for dichotomous outcomes were calculated by applying the pooled odds ratio to this baseline risk and are presented with corresponding 95% confidence intervals. For time-to-event outcomes (DFS and OS), effects are presented as hazard ratios; absolute effects were not estimated because baseline survival at a fixed time point was not prespecified and was inconsistently reported.

All analyses were performed using Review Manager (RevMan Web, Cochrane Collaboration). The protocol was registered in PROSPERO after commencement of the review (CRD420251266523; registered 1 December 2025). The review question, eligibility criteria, and co-primary outcomes were prespecified prior to data extraction.

## Results

### Study selection and characteristics

Four randomised controlled trials met the eligibility criteria [13–16], enrolling adults with mid or low rectal adenocarcinoma and comparing robotic with laparoscopic total mesorectal excision (Fig. 1). Across these studies 1,952 patients were randomised, with 977 assigned to robotic surgery and 975 to laparoscopy. Three trials randomised sphincter-preserving TME in mid or low rectum [13–15], and one trial randomised abdominoperineal resection for low rectal cancer [16]. Tumour height thresholds were within 10 cm from the anal verge. Pathology assessment was audited or assessor-blinded in the TME trials. Use of neoadjuvant therapy and baseline characteristics were broadly balanced between groups within each trial. Full study characteristics are depicted in Table 1.

**Table 1:** Characteristics of randomised controlled trials comparing robotic and laparoscopic total mesorectal excision for mid and low rectal adenocarcinoma. Abbreviations: APR, abdominoperineal resection; CRT, chemoradiotherapy; LAR, low anterior resection; MRF, mesorectal fascia; Nx, nodal status not specified in eligibility criteria; TME, total mesorectal excision

Study	Setting	Patients analysed (robotic vs. lap)	Key tumour inclusion criteria	Procedures	Neoadjuvant therapy (robotic vs. lap)	Robotic platform
REAL 2025	Multicentre, China	586 vs. 585	Rectal adenocarcinoma ≤ 10 cm from the anal verge; cT1–T3 with mesorectal fascia not involved, N0–N1, or ycT1–T3 Nx after preoperative radiotherapy or chemoradiotherapy; M0.	Sphincter-preserving resection (LAR + ISR): Robotic 487/586 (83.1%) vs. Laparoscopic 452/585 (77.3%) APR: Robotic 99/586 (16.9%) vs. Laparoscopic 133/585 (22.7%)	254/586 (43.3%) vs. 257/585 (43.9%). Preoperative radiotherapy or chemoradiotherapy permitted but not protocol-mandated (long-course only if given).	da Vinci Si Surgical System (Intuitive Surgical).
COLRAR 2023	Multicentre, South Korea	151 vs. 144	Rectal adenocarcinoma ≤ 10 cm from anal verge; cT1–4a, any N (Nx), M0	Sphincter-preserving resection (LAR + ISR): Robotic 123/151 (81.5%) vs. Laparoscopic 112/144 (77.8%) APR: Robotic 9/151 (6.0%) vs. Laparoscopic 14/144 (9.7%)	76/151 (50.3%) vs. 68/144 (47.2%). Preoperative chemoradiotherapy was permitted but not protocol-mandated; randomisation was stratified by receipt of CRT.	da Vinci Surgical System (Intuitive Surgical)
Kim 2018	Single centre, South Korea	66 vs. 73	Rectal adenocarcinoma located within 9 cm of the anal verge; clinical stage cT1–3, Nx, M0.	Sphincter-preserving resection (LAR + ISR): Robotic 65/66 (98.5%) vs. Laparoscopic 70/73 (95.9%) APR: Robotic 1/66 (1.5%) vs. Laparoscopic 2/73 (2.7%)	51/66 (77.3%) vs. 58/73 (79.5%). Preoperative chemoradiotherapy was administered for cT3 tumours according to institutional protocol; randomisation was stratified by receipt of CRT.	da Vinci S Surgical System (Intuitive Surgical)
Feng 2022	Single centre, China	174 vs. 173	Rectal adenocarcinoma ≤ 5 cm from the anal verge; clinical stage cT1–T3, N0–1 (or ycT1–T3 Nx after neoadjuvant chemoradiotherapy); M0; mesorectal fascia not involved.	APR: Robotic 174/174 (100%) vs. Laparoscopic 173/173 (100%)	37/174 (21.3%) vs. 35/173 (20.2%). Preoperative chemoradiotherapy permitted but not mandated; long-course CRT only if administered.	da Vinci S Surgical System (Intuitive Surgical)

### Risk of bias and certainty of evidence

Risk of bias was assessed at the outcome level using RoB 2.0. For the co-primary pathological outcomes (CRM positivity and mesorectal quality), overall risk of bias was low or showed some concerns, reflecting largely objective outcome measurement and, in several trials, blinded or audited pathological assessment. For perioperative outcomes (conversion and complications), overall risk of bias was more frequently judged as some concerns due to the unblinded nature of surgical trials and variability in outcome ascertainment and reporting. Outcome-level domain judgements are provided in Supplementary Fig. 1. Across outcomes, ‘some concerns’ were most commonly driven by deviations from intended interventions and outcome measurement in unblinded surgical trials, and by selection of the reported result where prespecification of analyses was unclear.

A Summary of Findings table (Table 2) was prepared in GRADEpro GDT, reporting absolute effects per 1,000 patients with corresponding 95% confidence intervals. Minimal important differences (MIDs) were prespecified a priori. For dichotomous outcomes, the MID was defined on the absolute scale as 2% for CRM positivity, 3% for complete TME, 2% for conversion, 3% for intraoperative complications, 5% for postoperative complications, and 2% for 3-year locoregional recurrence. For time-to-event outcomes (overall and disease-free survival), the MID was defined as a hazard ratio of 0.85. Certainty was downgraded for imprecision when the 95% confidence interval included both no important difference and an effect exceeding the prespecified MID.

**Table 2:** GRADE summary of findings for the primary outcomes in randomised trials of robotic versus laparoscopic total mesorectal excision for mid and low rectal cancer

Outcomes	Anticipated absolute effects^*^ (95% CI)		Relative effect (95% CI)	№ of participants (studies)	Certainty of the evidence (GRADE)	Comments
	Risk with Laparoscopic TME	Risk with Robotic TME				
CRM Positivity	66 per 1000	**39 per 1000** (26 to 58)	**OR 0.58** (0.38 to 0.87)	1896 (4 RCTs)	⨁⨁⨁⨁ High	Robotic TME was associated with lower CRM positivity.
Complete TME	883 per 1000	**921 per 1000** (896 to 941)	**OR 1.55** (1.14 to 2.12)	1952 (4 RCTs)	⨁⨁⨁⨁ High	Robotic TME was associated with higher TME completeness.
Conversion	31 per 1000	**13 per 1000** (7 to 24)	**OR 0.41** (0.21 to 0.79)	1952 (4 RCTs)	⨁⨁⨁⨁ High	Robotic TME was associated with lower conversion rates.
Intraoperative complications	77 per 1000	**56 per 1000** (37 to 84)	**OR 0.71** (0.46 to 1.10)	1952 (4 RCTs)	⨁⨁⨁◯ Moderate^a^	Robotic TME may reduce intraoperative complications, but the estimate is imprecise and compatible with no difference.
Postoperative complications	204 per 1000	**222 per 1000** (122 to 371)	**OR 1.11** (0.54 to 2.29)	1605 (3 RCTs)	⨁⨁◯◯ Low^b, c^	Robotic TME may make little to no difference to postoperative complications; the estimate is imprecise.
3 year local recurrence	42 per 1000	**19 per 1000** (10 to 34)	**OR 0.43** (0.23 to 0.81)	1517 (2 RCTs)	⨁⨁⨁◯ Moderate^d^	Robotic TME may reduce 3-year locoregional recurrence
Overall survival			**HR 0.79** (0.57 to 1.11) [Overall survival]	1517 (2 RCTs)	⨁⨁⨁◯ Moderate^e^	Robotic TME may make little to no difference to overall survival at current follow-up
3 year Disease Free Survival			**HR 0.78** (0.61 to 0.99) [3 year Disease Free Survival]	1517 (2 RCTs)	⨁⨁⨁◯ Moderate^f^	Robotic TME may improve 3-year disease-free survival, but the effect is small and imprecise

*The risk in the intervention group (and its 95% confidence interval) is based on the assumed risk in the comparison group and the **relative effect** of the intervention (and its 95% CI)

**CI**: confidence interval; **HR**: hazard ratio; **OR**: odds ratio

^a^Imprecision (serious): The pooled estimate is imprecise (OR 0.71, 95% CI 0.46–1.10). The CI crosses 1 and includes effects consistent with a clinically important reduction as well as no important difference. Given limited event numbers and a wide CI, we downgraded one level for imprecision

^b^Inconsistency (serious): There was substantial between-study heterogeneity in the effect estimate (I² = 78%), with variability in magnitude and direction across trials. Differences in definitions/ascertainment of “postoperative complications” and case mix may explain the inconsistency. We therefore downgraded one level for inconsistency

^c^Imprecision (serious): The pooled estimate is imprecise (OR 1.11, 95% CI 0.54–2.29). The confidence interval is wide, crosses the line of no effect, and includes both a clinically important reduction and a clinically important increase in complications. Event numbers were limited, so we downgraded one level for imprecision

^d^Imprecision (serious): Only two trials contributed data and the total number of locoregional recurrence events was low, resulting in limited information size. Although the pooled estimate suggests benefit (OR 0.43, 95% CI 0.23–0.81), the overall event rate is small and the confidence interval remains compatible with a smaller effect. We therefore downgraded one level for imprecision

^e^Imprecision (serious): The pooled hazard ratio is compatible with both a clinically important survival benefit and no important difference (HR 0.79, 95% CI 0.57–1.11). The confidence interval crosses 1.0 and the number of contributing trials and deaths is limited, so we downgraded one level for imprecision

^f^Imprecision (serious): Although the pooled estimate marginally favours robotic TME, the confidence interval is close to no effect (HR 0.78, 95% CI 0.61–0.99) and only two trials contributed data with limited events. The magnitude of benefit is therefore uncertain, so we downgraded one level for imprecision

### Primary outcomes

**Fig. 2:**
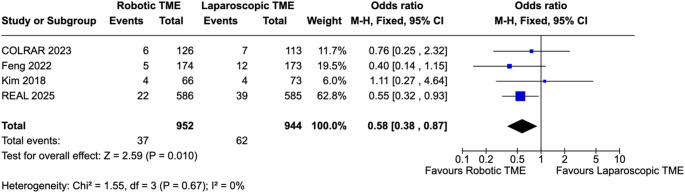
Pooled analysis of CRM positivity

**Fig. 3:**
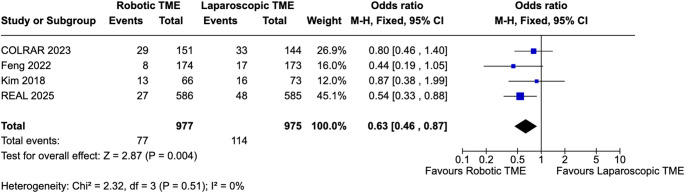
Pooled analysis of incomplete TME

Across the four included trials, robotic TME resulted in significantly improved pathological outcomes compared with laparoscopic TME. CRM positivity (Fig. 2) was significantly lower with robotic surgery, corresponding to a pooled odds ratio of 0.58 (95% CI 0.38–0.87; I² = 0%; *p* = 0.01). Mesorectal quality similarly favoured robotics (Fig. 3). The proportion of incomplete TME (nearly complete or incomplete) specimens was significantly higher in the laparoscopic group, yielding an OR of 0.63, 95% CI 0.46–0.87; I² = 0%; *p* = 0.004. When analysed inversely as complete TME, robotic surgery showed higher complete specimens; odds ratio of 1.55 (95% CI 1.14–2.12; I² = 0%; *p* = 0.006) (Supplementary Fig. 3). These effects were consistent across studies, with negligible statistical heterogeneity, indicating stable and directionally aligned improvements in resection quality with robotic TME. Sensitivity analysis excluding the APR-only trial did not materially change the pooled estimates for CRM positivity or complete TME, and the direction and statistical significance of effects were unchanged.

### Key secondary outcomes

**Fig. 4:**
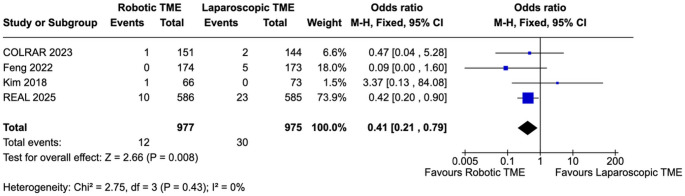
Pooled analysis of conversion

Conversion to open surgery occurred less frequently with robotic TME, yielding a pooled odds ratio of 0.41 (95% CI 0.21–0.79; I² = 0%; *p* = 0.008) (Fig. 4). Rates of intraoperative (OR 0.71, 95% CI 0.46–1.10; I² = 14%; *p* = 0.13) and postoperative complications (OR 1.11, 95% CI 0.54–2.29; I² = 78%; *p* = 0.77) were not significantly different between both cohorts (Supplementary Figs. 4–5).

### Long-term oncologic outcomes

**Fig. 5:**
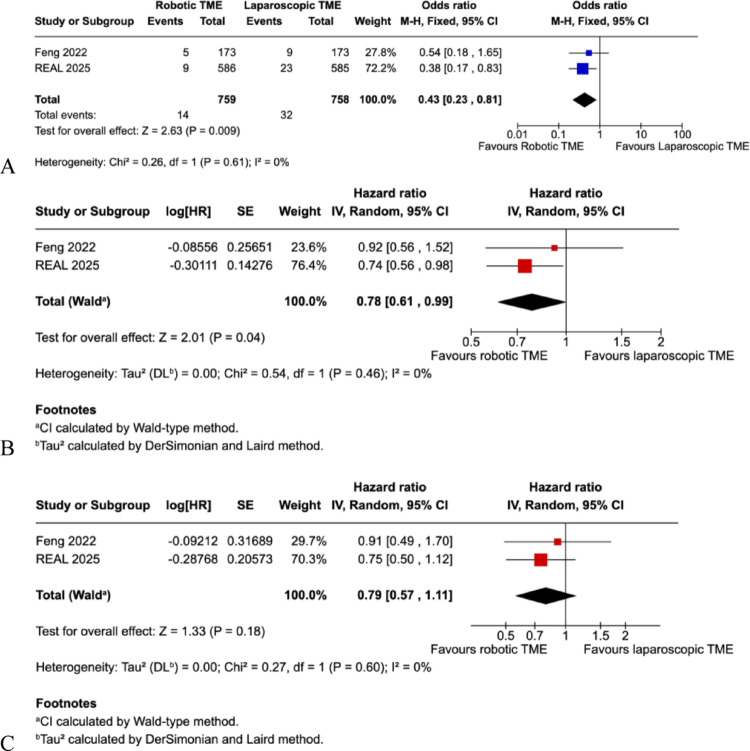
**A**: Pooled 3-year local recurrence rate. **B**: Pooled 3-year disease free survival. **C**: Pooled overall survival

Two trials reported three-year oncologic outcomes. Locoregional recurrence at three years was significantly lower in the robotic group compared to laparoscopic group (1.8% vs. 4.2%; OR 0.43, 95% CI 0.23–0.81; I² = 0%; *p* = 0.009) (Fig. 5A). Disease-free survival at three years also favoured robotics, with a pooled hazard ratio of 0.78 (95% CI 0.61–0.99; I² = 0%; *p* = 0.04) (Fig. 5B). Overall survival showed no significant difference between groups (HR 0.79, 95% CI 0.57–1.11; I² = 0%; *p* = 0.18) (Fig. 5C).

## Discussion

This meta-analysis confined to mid and low rectal cancer demonstrates that robotic TME improves resection quality and reduces conversion compared to laparoscopy, with early oncologic outcomes suggesting improved locoregional control. Robotic TME significantly reduced CRM positivity (OR 0.58, 95% CI 0.38–0.88, *p* = 0.01) and increased the rate of complete TME specimens (OR 1.55, 95% CI 1.13–2.11, *p* = 0.006), both with little heterogeneity (I² = 0%). Conversion to open surgery was also less frequent (OR 0.43, 95% CI 0.22–0.85, *p* = 0.02), a 1.9% absolute reduction, without an associated increase in intraoperative or short-term complications.

These technical gains are clinically meaningful. CRM involvement is a recognised driver of local recurrence and complete mesorectal excision reflects oncologic adequacy of the pelvic dissection [2, 22]. Consistent with this mechanism, the two trials [15, 16] reporting time-to-event outcomes showed an association with lower three-year locoregional recurrence with robotics (OR 0.43, 95% CI 0.23–0.81, *p* = 0.008) and a modest improvement in disease-free survival (HR 0.78, 95% CI 0.61–0.99, *p* = 0.04). Overall survival was similar at this follow-up (HR 0.79, 95% CI 0.57–1.11, *p* = 0.18), which is expected given low event rates and the time horizon and advances in systemic therapy. However, these oncologic findings are based on two studies with low event rates and should be interpreted as exploratory rather than confirmatory.

Although relative effects for CRM positivity and complete TME were statistically significant, absolute differences were modest because baseline event rates were low in these trials. In high-volume expert centres where CRM positivity is already uncommon and pathology audit is routine, small absolute gains may have limited impact on patient-level outcomes, particularly alongside contemporary neoadjuvant and systemic therapies. The clinical value of robotics is therefore likely to be context dependent, with the greatest potential benefit in technically demanding dissections (for example, very low tumours, threatened planes, narrow male pelvis, obesity, bulky disease, or after neoadjuvant therapy) where marginal improvements in plane fidelity and avoidance of conversion may be most consequential.

Unlike prior meta-analyses that combined tumour heights or included observational cohorts, or prioritised perioperative surrogates this review isolates the anatomical context in which robotics is most likely to influence outcomes [11, 23]. By restricting to randomised trials of mid and low tumours and anchoring the analysis on standardised histopathological outcomes, this synthesis isolates the setting in which robotics should matter most. The effect sizes were consistent across trials with I² = 0% for both co-primary outcomes, strengthening the inference that benefits are not centre-specific or platform-specific.

Despite low statistical heterogeneity, clinically relevant heterogeneity exists across included trials. One RCT evaluated abdominoperineal resection exclusively, whereas others included predominantly sphincter-preserving resections. Tumour height thresholds varied (very low tumours in some trials versus inclusion up to 10 cm in others), and neoadjuvant therapy and eligibility criteria relating to threatened mesorectal fascia were not uniformly standardised, potentially influencing baseline operative complexity and CRM risk. In addition, trials were largely conducted in high-volume centres with established minimally invasive expertise, which may affect conversion risk and pathological outcomes and limit generalisability to lower-volume settings.

Generalisability is also an important consideration. Three of four trials were conducted in East Asia in selected cohorts and predominantly high-volume centres with established minimally invasive expertise. Western rectal cancer populations typically include higher rates of obesity and comorbidity, and case mix, credentialing, and perioperative pathways may differ, all of which can influence technical difficulty, conversion risk, and postoperative outcomes. The absolute benefits observed here may not translate uniformly to lower-volume centres or settings without structured proctoring and routine pathological audit. Conversely, the relative advantage of robotics may be greater in anatomically challenging cases that are more prevalent in Western practice. Implementation outside trial settings should therefore be accompanied by careful case selection and monitoring of key quality metrics, including CRM positivity, TME grade, and conversion.

While robotic surgery has transformed the minimally invasive surgical landscape globally, access remains limited in countries such as Australia and New Zealand. This is largely attributable to the structure of the health system, with a predominant public sector operating under fixed budgets, competing service demands, and constrained capital and theatre resources [24]. In this context, universal adoption of robotic TME is neither feasible nor currently justifiable. Although an idealised model might favour robotic platforms for all rectal resections, real-world implementation requires careful prioritisation [25].

Learning curve and platform evolution may also influence observed effects. The included trials span a period of substantial change in robotic systems, instrumentation, imaging, and theatre integration, alongside increasing institutional experience with robotic pelvic dissection. It is plausible that earlier trials underestimate current robotic performance, particularly for deep pelvic plane control. At the same time, laparoscopic rectal surgery has also advanced over this period, potentially narrowing differences between approaches. Because trial reports variably described credentialing thresholds, surgeon case volumes, and platform generation, we could not perform meaningful subgroup analyses by experience or technology era. Future trials should report surgeon experience metrics and platform details alongside audited pathology and longer-term oncologic endpoints.

Within the practical constraints of healthcare systems, the present findings indicate that, on average across randomised trials in mid and low rectal cancer, robotic TME is associated with improved pathological resection quality and lower conversion rates. The included trials did not prespecify or report subgroup effects by pelvic anatomy, obesity, threatened CRM/mesorectal fascia, or neoadjuvant therapy exposure. Accordingly, any inference that benefits are greater in specific “high-difficulty” anatomical scenarios should be regarded as hypothesis-generating rather than established by the randomised evidence [10, 15, 16]. Robotic platforms may nonetheless offer technical and human-factor benefits, including stable three-dimensional visualisation, wristed instrumentation, motion scaling, and improved surgeon ergonomics [26]. Experimental and clinical data suggest that robotics reduces physical strain and cognitive workload compared with both open and laparoscopic surgery, potentially enhancing performance in prolonged and technically demanding pelvic dissections [27]. Given higher operative time and resource utilisation, implementation should be accompanied by service-level planning and routine auditing of pathological outcomes [9, 10, 24]. Such an approach maximises the likelihood that the technical and ergonomic advantages of robotic TME translate into meaningful oncologic benefit within resource-constrained health systems.

This review has strengths that increase confidence in the conclusions. It uses only randomised evidence, confines eligibility to tumours below the peritoneal reflection, prespecifies standardised pathology as co-primary outcomes, and includes maturing three-year oncologic endpoints that link technique with durability. Stoma-related outcomes could not be synthesised due to heterogeneous and incomplete reporting across trials. The APR-only trial inherently mandated a permanent stoma, and in the sphincter-preserving trials diversion and reversal were variably reported with inconsistent definitions and follow-up. Standardised, time-defined reporting of stoma endpoints should be incorporated into future rectal cancer surgical trials.

Future research should standardise definitions of CRM involvement and TME grading, adopt centralised pathology review, and report longer-term oncologic outcomes. Trials stratified by tumour height, neoadjuvant regimen, and pelvic morphology are also needed to identify the subgroups with greatest benefit. Economic evaluations will be essential to guide equitable implementation.

## Conclusion

In mid and low rectal cancer, robotic TME was associated with improved pathological resection quality and lower conversion rates compared with laparoscopic TME, without clear differences in early perioperative morbidity. Evidence for three-year oncologic outcomes is limited to two trials with few events; robotic TME was associated with lower three-year locoregional recurrence and a modest improvement in disease-free survival, while overall survival did not differ at current follow-up.

## Supplementary Information

Below is the link to the electronic supplementary material.

Supplementary Material 1

Supplementary Material 2

Supplementary Material 3

## Data Availability

No datasets were generated or analysed during the current study.
