# Supplementary material for: Resection quality and oncologic outcomes after robotic versus laparoscopic total mesorectal excision for mid and low rectal cancer: a systematic review and meta-analysis of randomised trials
Source: J Robot Surg. 2026 Jun 1;20(1):561. doi: 10.1007/s11701-026-03541-z (PMC13226396; doi:10.1007/s11701-026-03541-z)
Supplement: Supplementary file 1 — Supplementary Material 1 [file 11701_2026_3541_MOESM1_ESM.docx]

| **Trial** | **TME grading framework reported** | **Who graded TME quality** | **Blinding of pathology assessors** | **Centralised pathology review or audit/quality assurance protocol** |
| --- | --- | --- | --- | --- |
| REAL 2025 | Nagtegaal and Quirke | Hospital pathologists at each participating centre | Yes | Not centralised. Surgical quality oversight included mandatory intraoperative photos and unedited videos uploaded for review (plus pre-trial video submission by surgeons). |
| COLRAR 2023 | Nagtegaal and Quirke | Pathologists and surgeons at each centre assessed TME grade; discussion describes a two-step approach with surgeon grading followed by confirmatory specialist pathologist review. | Not stated | Not centralised. QA processes included regular investigator meetings evaluating interobserver agreement using a photograph database; surgeons also reviewed each other’s videos and specimen photographs for standardisation and quality control. |
| Feng 2022 | Nagtegaal and Quirke | Hospital pathologists | Not stated | Not centralised. |
| Kim 2018 | Nagtegaal and Quirke | Hospital pathologists | Yes | Not stated |

Supplementary Table 1: Pathology assessment and TME grading methods.

| **Databases** | **search strategy** | **Number** |
| --- | --- | --- |
| 1. PubMed | "Rectal Neoplasms"[Mesh] OR rectal cancer* OR rectal neoplasm* OR rectal carcinoma* OR rectal adenocarcinoma  AND  "low" OR "lower” OR distal OR "mid" OR middle OR "mid-rectal" OR "low-rectal" OR "lower-rectal" OR "distal rect*" OR "mid rect*" OR "low rect*") AND (rectum OR rectal  OR "anal verge")  AND  "Total Mesorectal Excision" OR TME OR "mesorectal excision"  OR Proctectomy[Mesh] OR proctectom*  OR "abdominoperineal resection" OR APR  AND  "Robotic Surgical Procedures"[Mesh] OR robot* assist* OR robot* surg* OR "da Vinci"  AND  "Laparoscopy"[Mesh] OR laparoscop*  AND  randomized controlled trial[pt] OR controlled clinical trial[pt] OR random* OR trial OR randomly OR placebo  NOT (animals[mh] NOT humans[mh])  AND ("2000/01/01"[dp] : "3000"[dp]) | 8 |
| 1. Cochrane library | #1 MeSH descriptor: [Rectal Neoplasms] explode all trees  #2 (rectal cancer* OR rectal neoplasm* OR rectal carcinoma* OR rectal adenocarcinoma*)  #3 ( (low OR lower OR distal OR mid OR middle OR "mid-rectal" OR "low-rectal" OR "lower-rectal" OR "distal rect*" OR "mid rect*" OR "low rect*") NEAR/3 (rectum OR rectal))  OR "anal verge"  #4 MeSH descriptor: [Proctectomy] explode all trees  OR ("Total Mesorectal Excision" OR TME OR "mesorectal excision" OR proctectom* OR "abdominoperineal resection" OR APR)  #5 MeSH descriptor: [Robotic Surgical Procedures] explode all trees  OR (robot* NEAR/3 (assist* OR surg*) OR "da Vinci")  #6 MeSH descriptor: [Laparoscopy] explode all trees  OR laparoscop*  #7 (random* OR randomly OR trial OR placebo)  #8 (#1 OR #2) AND #3 AND #4 AND #5 AND #6 AND #7 | 20 |
| 1. Web of Science | TS=(rectal cancer* OR rectal neoplasm* OR rectal carcinoma* OR rectal adenocarcinoma*)  AND  TS=( ( (low OR lower OR distal OR mid OR middle OR "mid-rectal" OR "low-rectal" OR "lower-rectal" OR "distal rect*" OR "mid rect*" OR "low rect*")  NEAR/3 (rectum OR rectal) )  OR "anal verge" )  AND  TS=("total mesorectal excision" OR TME OR "mesorectal excision" OR proctectom* OR "abdominoperineal resection" OR APR)  AND  TS=( (robot* NEAR/3 (assist* OR surg*) ) OR "da Vinci" )  AND  TS=(laparoscop*)  AND  TS=(random* OR randomly OR trial*) | 210 |

Supplementary Table 2: Search strategy.
