# Supplementary material for: Resection quality and oncologic outcomes after robotic versus laparoscopic total mesorectal excision for mid and low rectal cancer: a systematic review and meta-analysis of randomised trials
Source: J Robot Surg. 2026 Jun 1;20(1):561. doi: 10.1007/s11701-026-03541-z (PMC13226396; doi:10.1007/s11701-026-03541-z)
Supplement: Supplementary file 2 — Supplementary Material 2 [file 11701_2026_3541_MOESM2_ESM.docx]

Supplementary Figure 1. Risk of bias (RoB 2.0) assessed at the outcome level. Domain-level judgements are shown for each included trial across RoB 2.0 domains: D1, bias arising from the randomisation process; D2, bias due to deviations from intended interventions; D3, bias due to missing outcome data; D4, bias in measurement of the outcome; D5, bias in selection of the reported result. Green indicates low risk and yellow indicates some concerns. Panels: A) CRM positivity; B) complete TME; C) conversion; D) intraoperative complications; E) postoperative complications; F) 3-year locoregional recurrence; G) 3-year disease-free survival; H) overall survival.


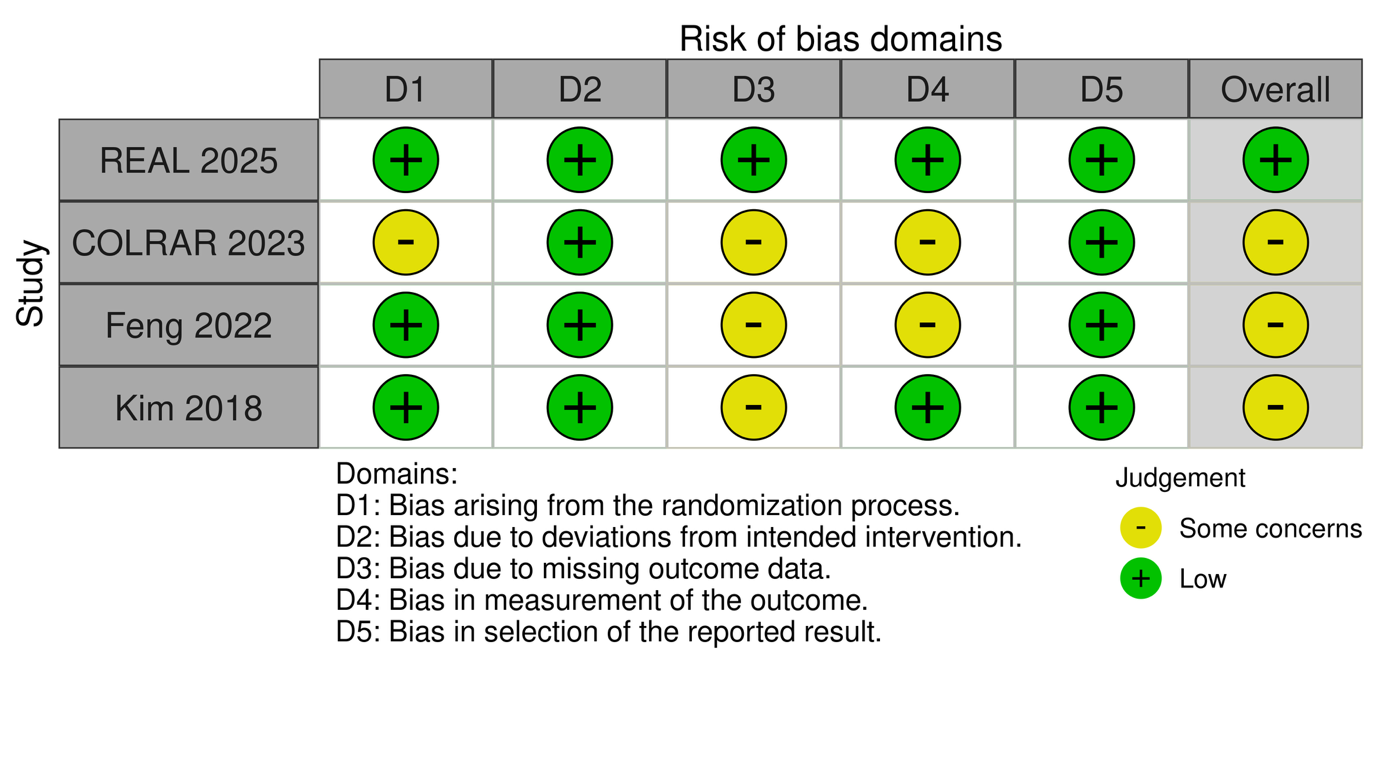


1. CRM positivity.


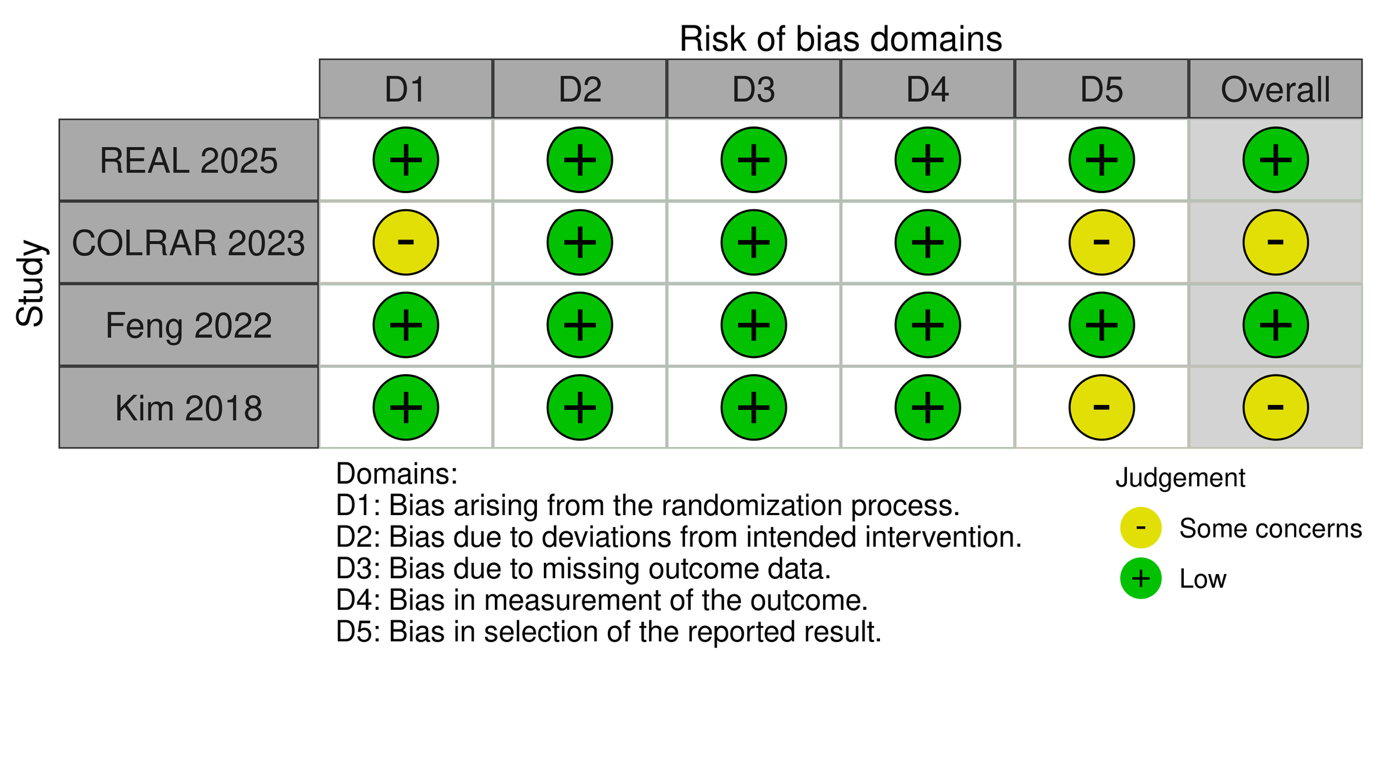


1. Complete TME


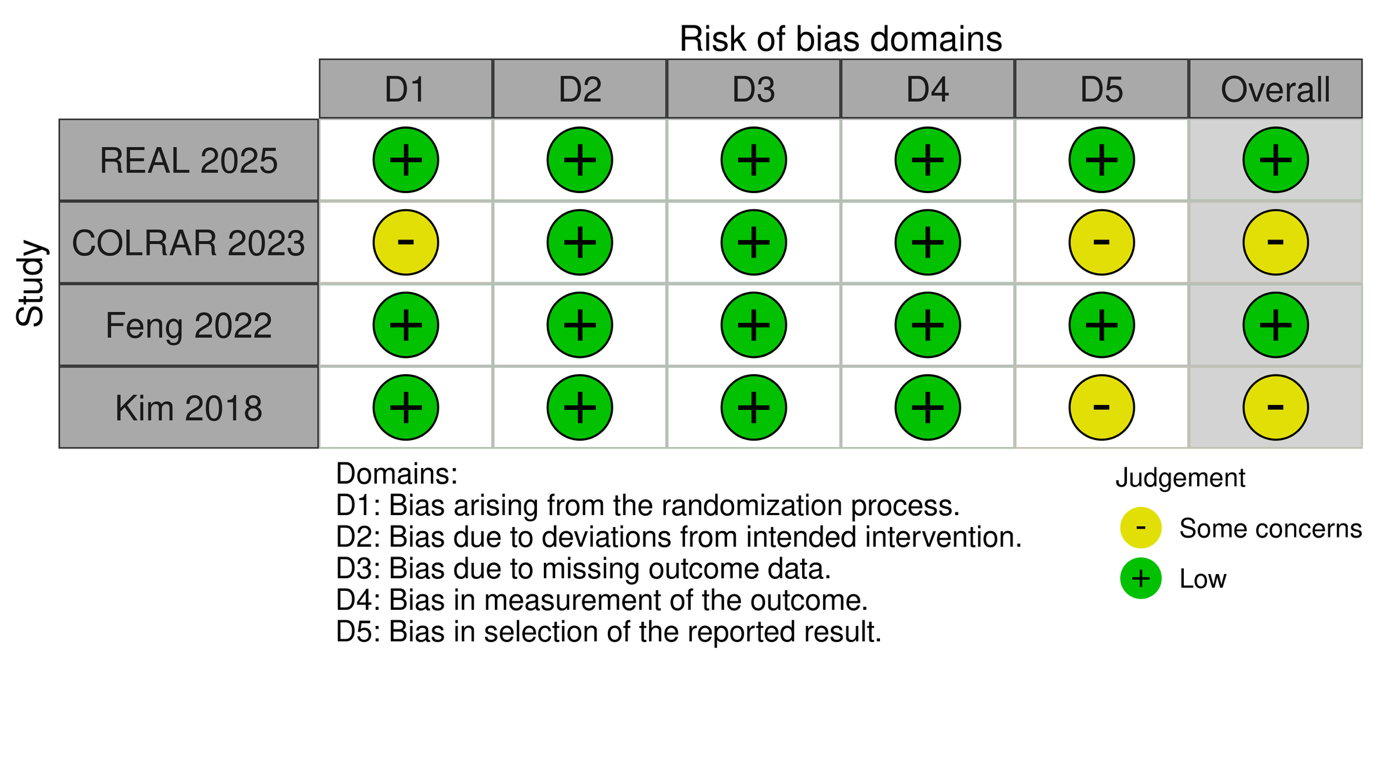


1. Conversion


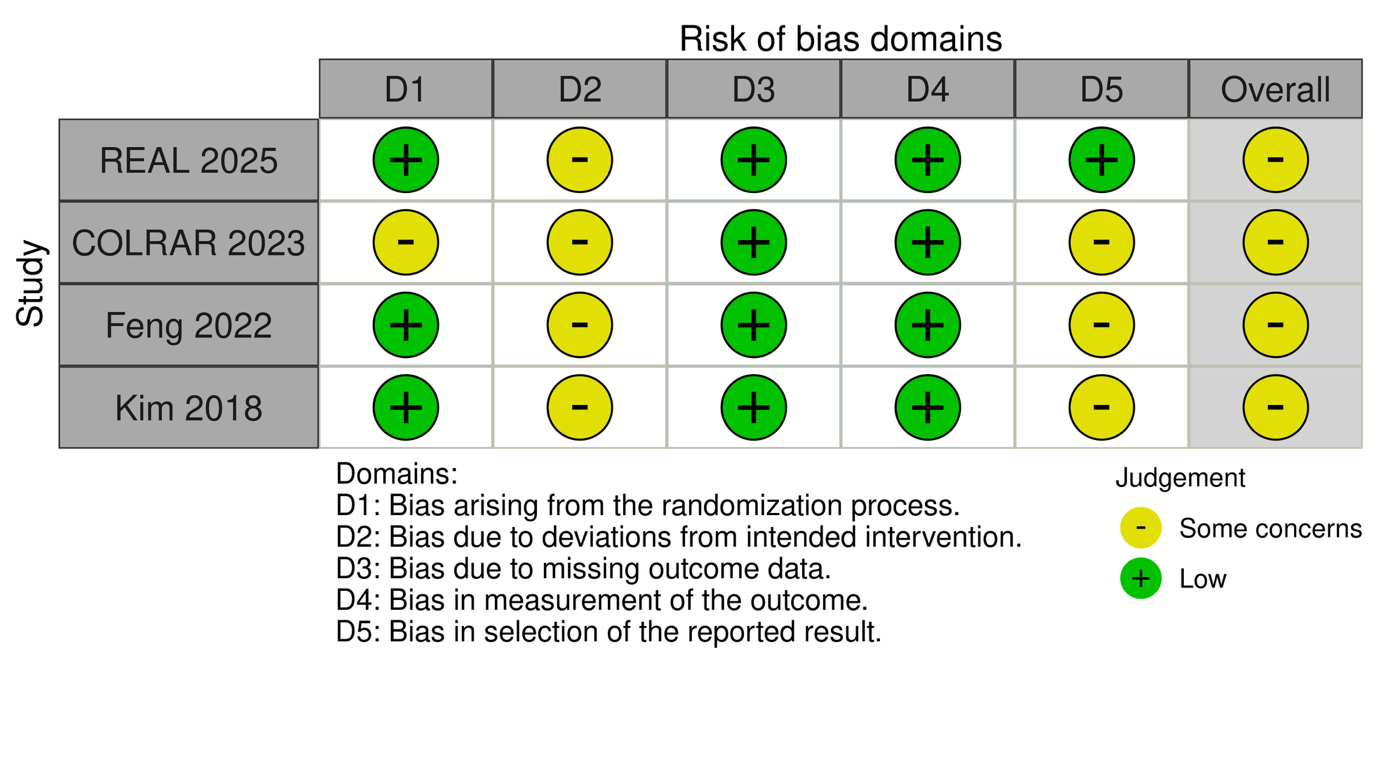


1. Intraoperative complications


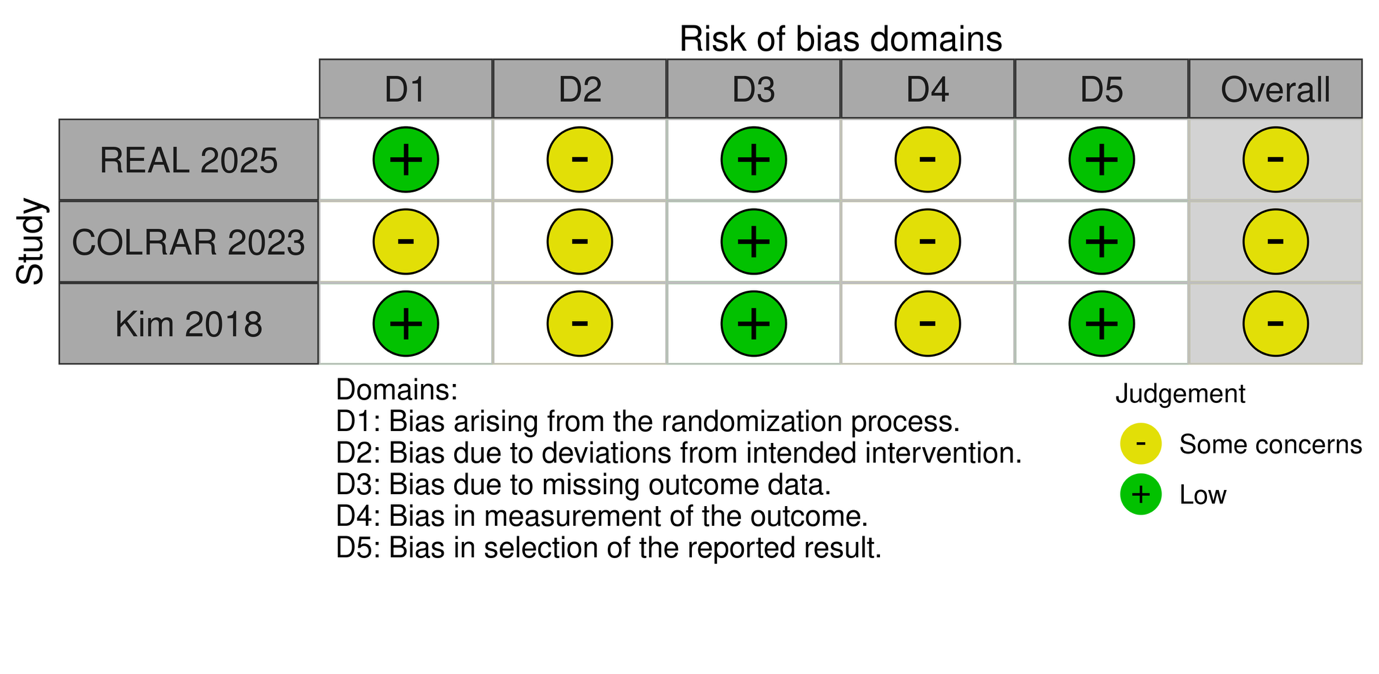


1. Postoperative complications.


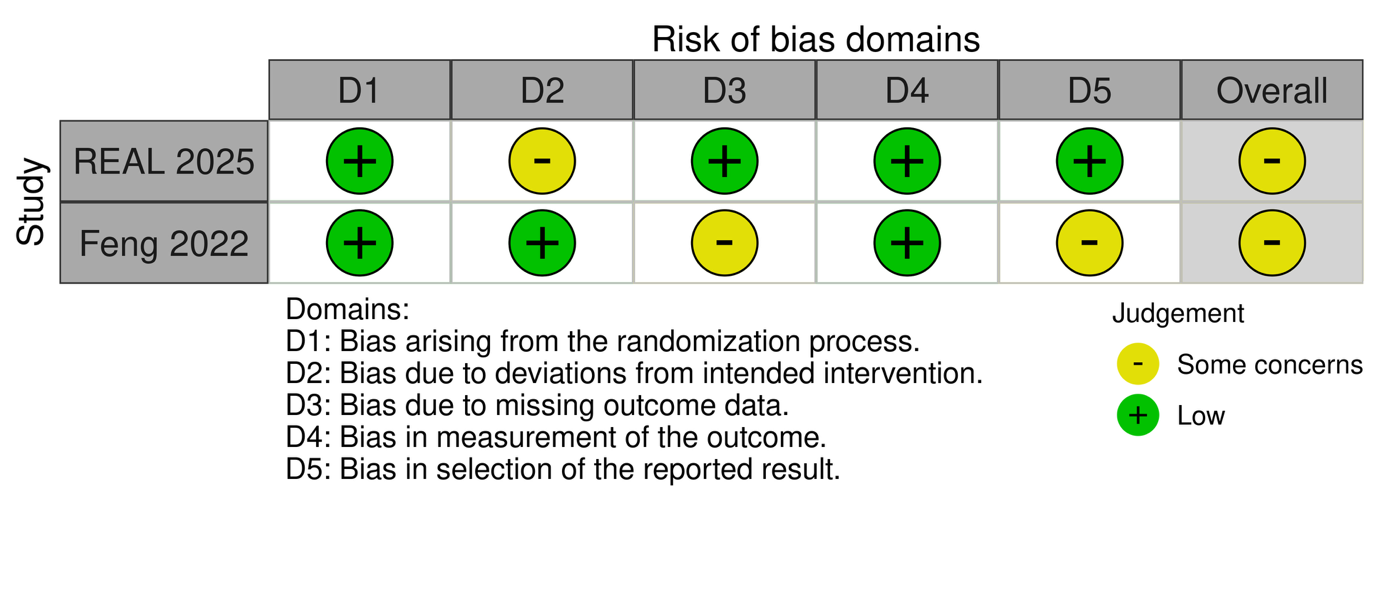


1. 3-year locoregional recurrence rate.


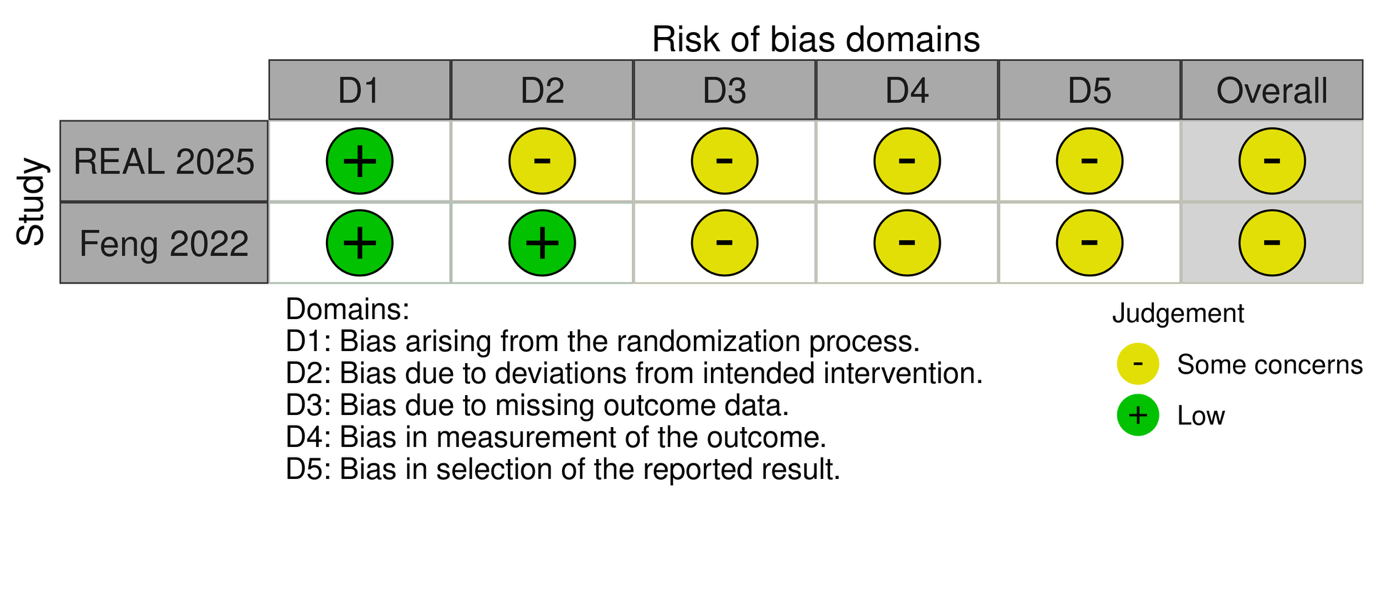


1. 3-year disease free survival.


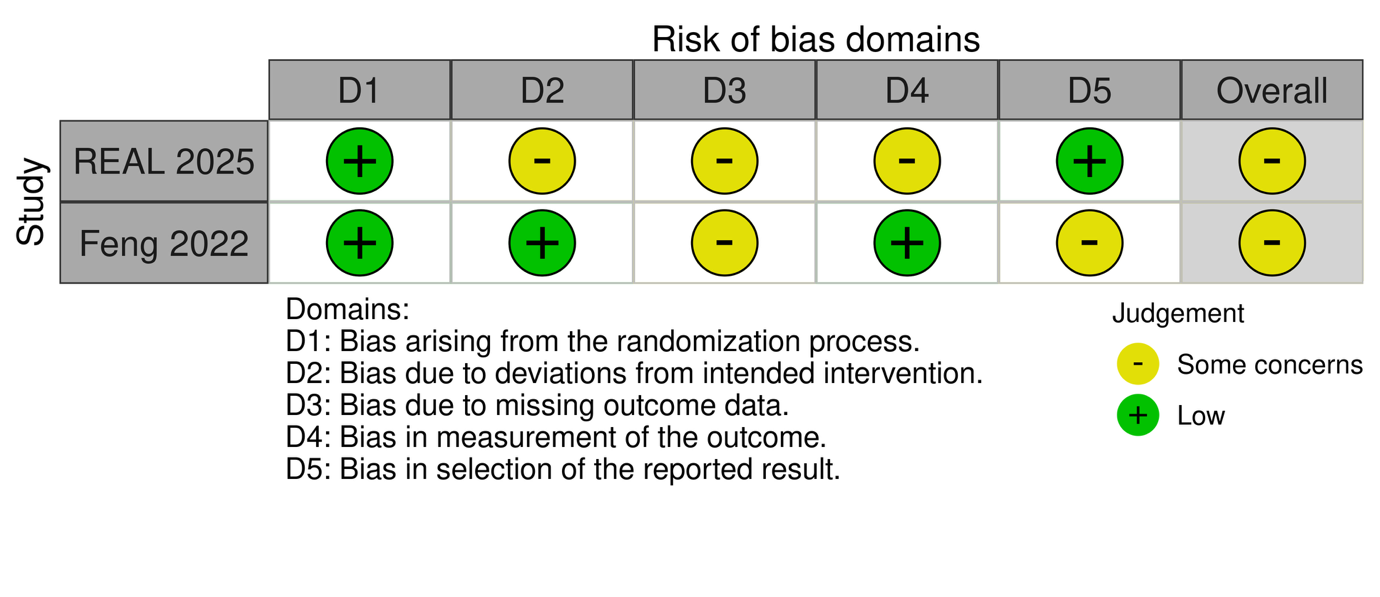


H) Overall survival.


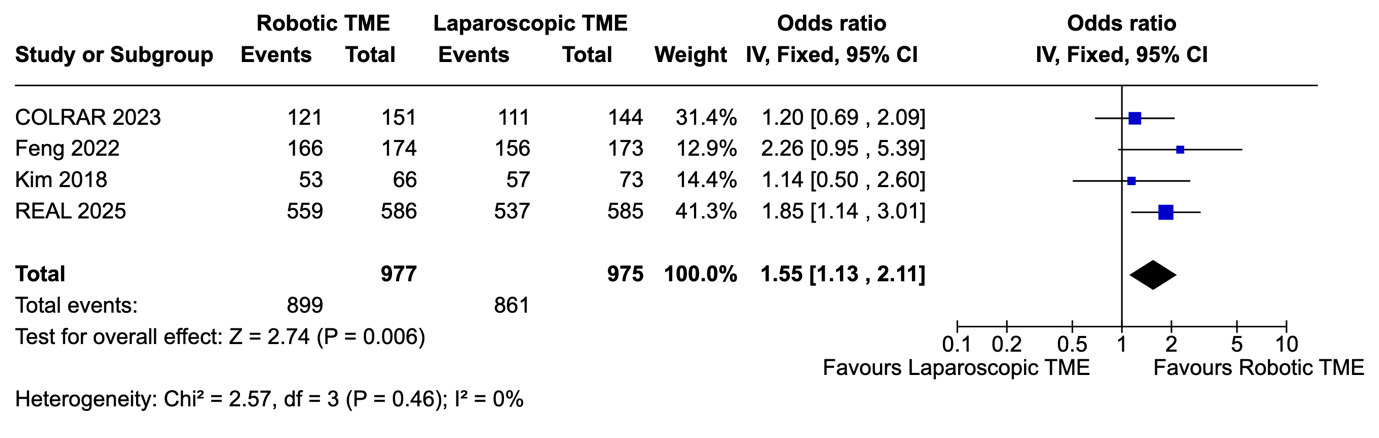


Supplementary Figure 3: Pooled analysis of complete TME.


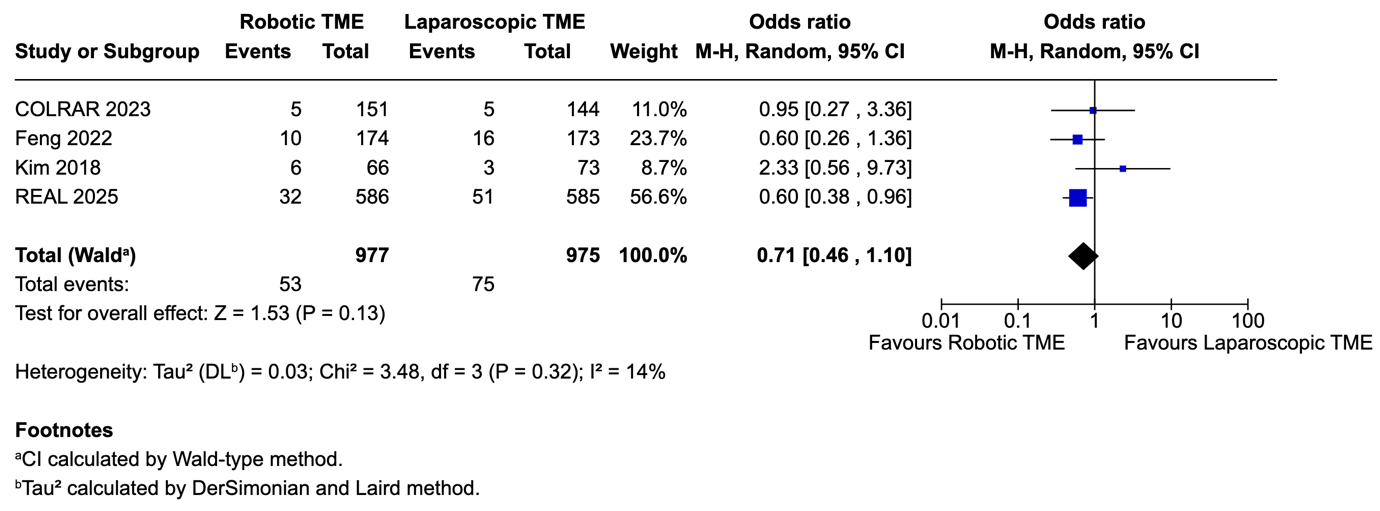


Supplementary Figure 4: Pooled intraoperative complications.


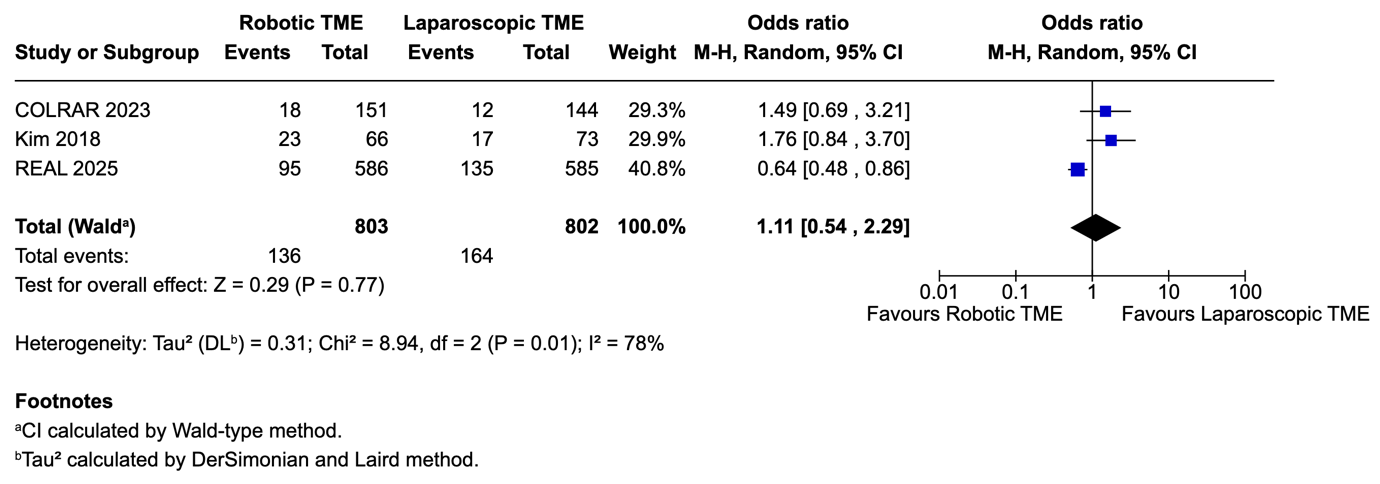


Supplementary Figure 5: Pooled postoperative complications.
